# Propan-2-yl 2-(1,1,3-trioxo-2,3-dihydro-1λ^6^,2-benzothia­zol-2-yl)acetate

**DOI:** 10.1107/S1600536812036148

**Published:** 2012-08-23

**Authors:** Muhammad Zia-ur-Rehman, Bilal Shahid, Hamid Latif Siddiqui, Tanveer Ahmad, Masood Parvez

**Affiliations:** a Applied Chemistry Research Centre, PCSIR Laboratories Complex, Lahore 54600, Pakistan; b Institute of Chemistry, University of the Punjab, Lahore 54590, Pakistan; c Department of Chemistry, The University of Calgary, 2500 University Drive NW, Calgary, Alberta, Canada T2N 1N4

## Abstract

In the title mol­ecule, C_12_H_13_NO_5_S, the benzisothia­zole ring system is essentially planar (r.m.s. deviation = 0.0169 Å) as is the –C—C(=O)—O—C– sequence of atoms in the vicinity of the acetate group (r.m.s. deviation = 0.0044 Å). The mean plane of these atoms forms a dihedral angle of 88.41 (7)° with the benzisothia­zole ring system. In the crystal, weak C—H⋯O hydrogen bonds involving methyl­ene and methyne H atoms form *R*_4_^3^(20) graph-set motifs.

## Related literature

For uses of 1,2-benzothia­zol-3(2*H*)-one 1,1-dioxide, see: Kap-Sun & Nicholas (1998 ▶). For the synthesis of non-steroidal anti-inflammatory drugs (NSAIDs) and their biological evaluation, see: Ahmad *et al.* (2011 ▶); Zia-ur-Rehman *et al.* (2009 ▶). For related structures, see: Sattar *et al.* (2012 ▶); Maliha *et al.* (2007 ▶); Siddiqui *et al.* (2007 ▶). For graph-set motifs, see: (Bernstein *et al.*, 1995 ▶).
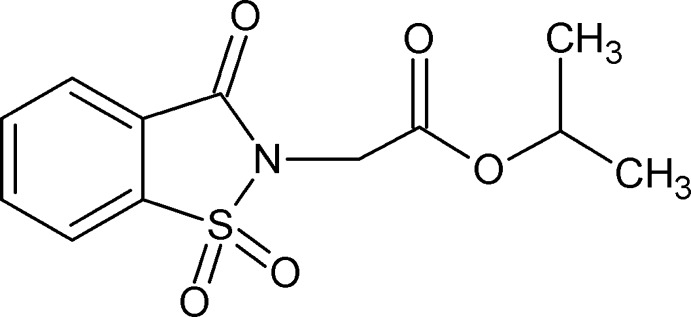


## Experimental

### 

#### Crystal data

C_12_H_13_NO_5_S
*M* *_r_* = 283.29
Monoclinic, 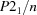

*a* = 8.0922 (3) Å
*b* = 9.2314 (4) Å
*c* = 17.7414 (8) Å
β = 100.075 (2)°
*V* = 1304.89 (9) Å^3^
*Z* = 4
Mo *K*α radiation
μ = 0.26 mm^−1^
*T* = 173 K
0.14 × 0.12 × 0.06 mm

#### Data collection

Nonius KappaCCD diffractometer
Absorption correction: multi-scan (*SORTAV*; Blessing, 1997 ▶) *T*_min_ = 0.964, *T*_max_ = 0.984
5518 measured reflections
2953 independent reflections
2339 reflections with *I* > 2σ(*I*)
*R*_int_ = 0.041

#### Refinement

*R*[*F* ^2^ > 2σ(*F* ^2^)] = 0.055
*wR*(*F* ^2^) = 0.119
*S* = 1.11
2953 reflections
174 parameters
H-atom parameters constrained
Δρ_max_ = 0.41 e Å^−3^
Δρ_min_ = −0.43 e Å^−3^

### 

Data collection: *COLLECT* (Hooft, 1998 ▶); cell refinement: *DENZO* (Otwinowski & Minor, 1997 ▶); data reduction: *SCALEPACK* (Otwinowski & Minor, 1997 ▶); program(s) used to solve structure: *SHELXS97* (Sheldrick, 2008 ▶); program(s) used to refine structure: *SHELXL97* (Sheldrick, 2008 ▶); molecular graphics: *ORTEP-3 for Windows* (Farrugia, 1997 ▶); software used to prepare material for publication: *SHELXL97*.

## Supplementary Material

Crystal structure: contains datablock(s) global, I. DOI: 10.1107/S1600536812036148/lh5516sup1.cif

Structure factors: contains datablock(s) I. DOI: 10.1107/S1600536812036148/lh5516Isup2.hkl

Supplementary material file. DOI: 10.1107/S1600536812036148/lh5516Isup3.cml

Additional supplementary materials:  crystallographic information; 3D view; checkCIF report

## Figures and Tables

**Table 1:** Hydrogen-bond geometry (Å, °)

*D*—H⋯*A*	*D*—H	H⋯*A*	*D*⋯*A*	*D*—H⋯*A*
C8—H8*B*⋯O3^i^	0.99	2.27	3.236 (3)	166
C10—H10⋯O3^ii^	1.00	2.42	3.245 (3)	140

Symmetry codes: (i) 
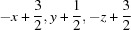
; (ii) 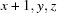.

**Fig. 1.**
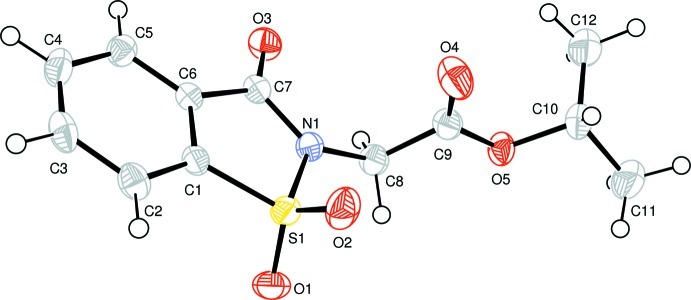
The molecular structure of the title compound with displacement ellipsoids drawn at the 50% probability level. H atoms are shown as small spheres of arbitrary radius.

**Fig. 2.**
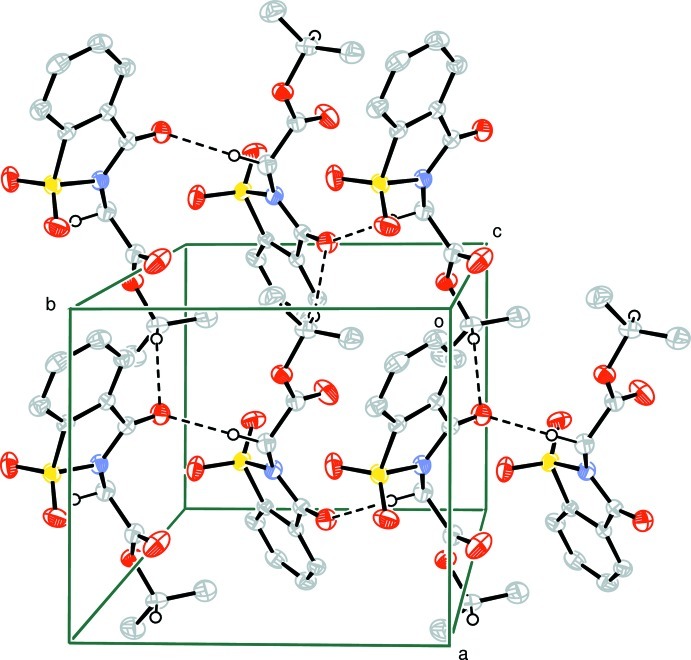
A part of the crystal structure showing the C—H···O hydrogen bonds (dotted lines) forming R43(20) graph set motifs. H atoms not involved in hydrogen bonds are omitted for clarity.

## supplementary crystallographic information

### Comment

1,2-Benzothiazol-3(2*H*)-one 1,1-dioxide is known as an artificial sweetener commonly known as saccharin. It has been widely explored as a reactant of a number of medicinally important heterocyclic compounds (Kap-Sun & Nicholas, 1998), out of which oxicam family is the most important. *N*-alkylation of saccharin followed by base catalyzed ring expansion gives rise to methyl 4-hydroxy-2*H*-1,2-benzothiazine-3-carboxylate 1,1-dioxide (Zia-ur-Rehman *et al.*, 2009) which is a basic precursor to the synthesis of Piroxicam, Meloxicam and Ampiroxicam. In continuation of our work on the synthesis and biological evaluation of thiazine based compounds (Ahmad *et al.*, 2011), we herein report the crystal structure of the title compound.

In the title compound (Fig. 1), the benzisothiazol ring system S1/N1/C1–C7 is essentially planar with an r.m.s. deviation of the fitted atoms being 0.0169 Å. The O4/O5/C8-C10 sequence of atoms is also planar (r.m.s. deviation = 0.0044 Å) and forms a dihedral angle of 88.41 (7)° with the mean plane of the benzisothiazole ring system. The crystal packing is consolidated by weak intermolecular C—H···O hydrogen bonding interactions involving a H-atom of the methylene C8, C8—H8B···O3^i^, and a methyne H-atom bound to C10, C10—H10···O3^ii^, forming twenty membered rings in graph set motif *R*_4_^3^(20) (Bernstein *et al.*, 1995) (Fig. 2 & Tab. 1).

The bond distances and angles in the title compound agree very well with the corresponding bond distances and angles reported in closely related compounds (Sattar *et al.*, 2012); Maliha *et al.*, 2007; Siddiqui *et al.*, 2007).

### Experimental

A mixture of sodium saccharin (7.50 g; 36.55 mmol), *N*,*N*-dimethylformamide (50 ml) and isopropyl chloroacetate (4.99 g; 36.55 mmol) was taken in a round bottom flask and immersed in ultrasonic reaction bath at 333 K for a period of 15 min. The contents were then cooled to room temperature and poured over ice cooled water (300 ml) resulting in the formation of the title compound as a white solid, which was filtered and washed with cold water. The product was dried and recrystallized from isopropyl alcohol by slow evaporation to yield the crystal suitable for single crystal X-ray diffraction, yield = 94.4%; m.p. 392–394 K.

### Refinement

All H atoms were positioned geometrically and refined using a riding model, with C—H = 0.95, 0.98 and 0.99 Å, for aryl, methyl and methylene H-atoms, respectively. The *U*_iso_(H) were allowed at 1.2*U*_eq_(C).

### Crystal data

**Table:**

C_12_H_13_NO_5_S	*F*(000) = 592
*M**_r_* = 283.29	*D*_x_ = 1.442 Mg m^−^^3^
Monoclinic, *P*2_1_/*n*	Mo *K*α radiation, λ = 0.71073 Å
Hall symbol: -P 2yn	Cell parameters from 2817 reflections
*a* = 8.0922 (3) Å	θ = 1.0–27.5°
*b* = 9.2314 (4) Å	µ = 0.26 mm^−^^1^
*c* = 17.7414 (8) Å	*T* = 173 K
β = 100.075 (2)°	Prism, colorless
*V* = 1304.89 (9) Å^3^	0.14 × 0.12 × 0.06 mm
*Z* = 4	

### Data collection

**Table:**

Nonius KappaCCD diffractometer	2953 independent reflections
Radiation source: fine-focus sealed tube	2339 reflections with *I* > 2σ(*I*)
Graphite monochromator	*R*_int_ = 0.041
ω and φ scans	θ_max_ = 27.4°, θ_min_ = 3.2°
Absorption correction: multi-scan (*SORTAV*; Blessing, 1997)	*h* = −10→10
*T*_min_ = 0.964, *T*_max_ = 0.984	*k* = −11→11
5518 measured reflections	*l* = −22→22

### Refinement

**Table:**

Refinement on *F*^2^	Primary atom site location: structure-invariant direct methods
Least-squares matrix: full	Secondary atom site location: difference Fourier map
*R*[*F*^2^ > 2σ(*F*^2^)] = 0.055	Hydrogen site location: inferred from neighbouring sites
*wR*(*F*^2^) = 0.119	H-atom parameters constrained
*S* = 1.11	*w* = 1/[σ^2^(*F*_o_^2^) + (0.0186*P*)^2^ + 1.8823*P*] where *P* = (*F*_o_^2^ + 2*F*_c_^2^)/3
2953 reflections	(Δ/σ)_max_ = 0.001
174 parameters	Δρ_max_ = 0.41 e Å^−^^3^
0 restraints	Δρ_min_ = −0.43 e Å^−^^3^

### Special details

**Table:**

Geometry. All e.s.d.'s (except the e.s.d. in the dihedral angle between two l.s. planes) are estimated using the full covariance matrix. The cell e.s.d.'s are taken into account individually in the estimation of e.s.d.'s in distances, angles and torsion angles; correlations between e.s.d.'s in cell parameters are only used when they are defined by crystal symmetry. An approximate (isotropic) treatment of cell e.s.d.'s is used for estimating e.s.d.'s involving l.s. planes.
Refinement. Refinement of *F*^2^ against ALL reflections. The weighted *R*-factor *wR* and goodness of fit *S* are based on *F*^2^, conventional *R*-factors *R* are based on *F*, with *F* set to zero for negative *F*^2^. The threshold expression of *F*^2^ > σ(*F*^2^) is used only for calculating *R*-factors(gt) *etc*. and is not relevant to the choice of reflections for refinement. *R*-factors based on *F*^2^ are statistically about twice as large as those based on *F*, and *R*- factors based on ALL data will be even larger.

### Fractional atomic coordinates and isotropic or equivalent isotropic displacement parameters (Å^2^)

**Table:**

	*x*	*y*	*z*	*U*_iso_*/*U*_eq_	
S1	0.71458 (8)	0.28520 (7)	0.59318 (4)	0.02941 (18)	
O1	0.6975 (3)	0.4281 (2)	0.62206 (12)	0.0414 (5)	
O2	0.8538 (2)	0.2573 (2)	0.55647 (11)	0.0423 (5)	
O3	0.5438 (2)	−0.0124 (2)	0.70065 (10)	0.0335 (4)	
O4	0.9985 (3)	−0.0077 (2)	0.68519 (12)	0.0488 (6)	
O5	1.0959 (2)	0.1079 (2)	0.79588 (10)	0.0311 (4)	
N1	0.7092 (3)	0.1656 (2)	0.66356 (12)	0.0277 (5)	
C1	0.5243 (3)	0.2216 (3)	0.54085 (13)	0.0250 (5)	
C2	0.4415 (3)	0.2730 (3)	0.47085 (14)	0.0321 (6)	
H2	0.4873	0.3475	0.4438	0.039*	
C3	0.2872 (3)	0.2088 (3)	0.44250 (15)	0.0362 (7)	
H3	0.2259	0.2407	0.3948	0.043*	
C4	0.2206 (3)	0.1002 (3)	0.48162 (15)	0.0350 (6)	
H4	0.1149	0.0594	0.4605	0.042*	
C5	0.3061 (3)	0.0497 (3)	0.55150 (15)	0.0293 (6)	
H5	0.2605	−0.0248	0.5786	0.035*	
C6	0.4602 (3)	0.1119 (3)	0.58023 (13)	0.0234 (5)	
C7	0.5697 (3)	0.0762 (3)	0.65414 (14)	0.0257 (5)	
C8	0.8296 (3)	0.1713 (3)	0.73484 (14)	0.0314 (6)	
H8A	0.7737	0.1390	0.7773	0.038*	
H8B	0.8656	0.2730	0.7451	0.038*	
C9	0.9831 (3)	0.0785 (3)	0.73385 (14)	0.0301 (6)	
C10	1.2566 (3)	0.0297 (3)	0.80553 (16)	0.0350 (6)	
H10	1.2916	0.0173	0.7546	0.042*	
C11	1.3818 (4)	0.1243 (4)	0.8560 (2)	0.0526 (9)	
H11A	1.4929	0.0790	0.8629	0.063*	
H11B	1.3475	0.1359	0.9060	0.063*	
H11C	1.3864	0.2195	0.8320	0.063*	
C12	1.2351 (4)	−0.1147 (4)	0.8396 (2)	0.0501 (8)	
H12A	1.3404	−0.1691	0.8444	0.060*	
H12B	1.1458	−0.1680	0.8065	0.060*	
H12C	1.2048	−0.1022	0.8903	0.060*	

### Atomic displacement parameters (Å^2^)

**Table:**

	*U*^11^	*U*^22^	*U*^33^	*U*^12^	*U*^13^	*U*^23^
S1	0.0286 (3)	0.0264 (3)	0.0310 (3)	−0.0029 (3)	−0.0007 (2)	0.0048 (3)
O1	0.0497 (12)	0.0242 (10)	0.0452 (12)	−0.0044 (9)	−0.0059 (9)	0.0021 (9)
O2	0.0295 (10)	0.0523 (13)	0.0462 (12)	−0.0056 (10)	0.0100 (9)	0.0067 (10)
O3	0.0376 (10)	0.0338 (10)	0.0291 (9)	0.0005 (9)	0.0057 (8)	0.0080 (8)
O4	0.0480 (13)	0.0531 (14)	0.0386 (11)	0.0179 (11)	−0.0107 (9)	−0.0165 (10)
O5	0.0254 (9)	0.0375 (11)	0.0275 (9)	0.0022 (8)	−0.0034 (7)	−0.0044 (8)
N1	0.0281 (11)	0.0263 (11)	0.0257 (11)	−0.0001 (9)	−0.0035 (9)	0.0036 (9)
C1	0.0239 (12)	0.0249 (12)	0.0253 (12)	0.0039 (10)	0.0019 (9)	−0.0003 (10)
C2	0.0324 (14)	0.0362 (15)	0.0280 (13)	0.0062 (12)	0.0057 (11)	0.0066 (12)
C3	0.0323 (14)	0.0452 (17)	0.0281 (13)	0.0119 (13)	−0.0035 (11)	0.0039 (13)
C4	0.0257 (13)	0.0436 (16)	0.0329 (14)	0.0012 (12)	−0.0027 (11)	−0.0058 (13)
C5	0.0258 (13)	0.0315 (14)	0.0309 (13)	0.0000 (11)	0.0063 (10)	−0.0037 (11)
C6	0.0241 (12)	0.0225 (12)	0.0235 (12)	0.0030 (10)	0.0039 (9)	0.0009 (10)
C7	0.0258 (12)	0.0255 (13)	0.0254 (12)	0.0030 (10)	0.0036 (10)	0.0005 (10)
C8	0.0332 (14)	0.0313 (14)	0.0259 (12)	0.0005 (11)	−0.0057 (11)	−0.0020 (11)
C9	0.0323 (14)	0.0310 (14)	0.0239 (12)	0.0017 (11)	−0.0033 (10)	−0.0005 (11)
C10	0.0240 (13)	0.0438 (16)	0.0369 (14)	0.0018 (12)	0.0047 (11)	−0.0011 (13)
C11	0.0359 (17)	0.055 (2)	0.061 (2)	−0.0069 (15)	−0.0066 (15)	0.0023 (17)
C12	0.0375 (17)	0.0480 (19)	0.062 (2)	0.0002 (15)	0.0017 (15)	0.0066 (17)

### Geometric parameters (Å, º)

**Table:**

S1—O2	1.420 (2)	C4—H4	0.9500
S1—O1	1.431 (2)	C5—C6	1.387 (3)
S1—N1	1.673 (2)	C5—H5	0.9500
S1—C1	1.754 (2)	C6—C7	1.485 (3)
O3—C7	1.206 (3)	C8—C9	1.511 (4)
O4—C9	1.197 (3)	C8—H8A	0.9900
O5—C9	1.329 (3)	C8—H8B	0.9900
O5—C10	1.471 (3)	C10—C12	1.486 (4)
N1—C7	1.385 (3)	C10—C11	1.508 (4)
N1—C8	1.456 (3)	C10—H10	1.0000
C1—C6	1.381 (3)	C11—H11A	0.9800
C1—C2	1.388 (3)	C11—H11B	0.9800
C2—C3	1.395 (4)	C11—H11C	0.9800
C2—H2	0.9500	C12—H12A	0.9800
C3—C4	1.381 (4)	C12—H12B	0.9800
C3—H3	0.9500	C12—H12C	0.9800
C4—C5	1.390 (4)		
			
O2—S1—O1	117.72 (13)	O3—C7—C6	127.4 (2)
O2—S1—N1	110.45 (12)	N1—C7—C6	108.8 (2)
O1—S1—N1	108.93 (12)	N1—C8—C9	113.3 (2)
O2—S1—C1	112.96 (12)	N1—C8—H8A	108.9
O1—S1—C1	111.56 (12)	C9—C8—H8A	108.9
N1—S1—C1	92.23 (11)	N1—C8—H8B	108.9
C9—O5—C10	117.5 (2)	C9—C8—H8B	108.9
C7—N1—C8	122.3 (2)	H8A—C8—H8B	107.7
C7—N1—S1	115.50 (16)	O4—C9—O5	126.2 (2)
C8—N1—S1	121.56 (18)	O4—C9—C8	125.1 (2)
C6—C1—C2	122.6 (2)	O5—C9—C8	108.7 (2)
C6—C1—S1	110.48 (17)	O5—C10—C12	108.9 (2)
C2—C1—S1	126.9 (2)	O5—C10—C11	105.9 (2)
C1—C2—C3	116.0 (3)	C12—C10—C11	113.1 (3)
C1—C2—H2	122.0	O5—C10—H10	109.6
C3—C2—H2	122.0	C12—C10—H10	109.6
C4—C3—C2	122.1 (2)	C11—C10—H10	109.6
C4—C3—H3	119.0	C10—C11—H11A	109.5
C2—C3—H3	119.0	C10—C11—H11B	109.5
C3—C4—C5	121.0 (2)	H11A—C11—H11B	109.5
C3—C4—H4	119.5	C10—C11—H11C	109.5
C5—C4—H4	119.5	H11A—C11—H11C	109.5
C6—C5—C4	117.7 (3)	H11B—C11—H11C	109.5
C6—C5—H5	121.2	C10—C12—H12A	109.5
C4—C5—H5	121.2	C10—C12—H12B	109.5
C1—C6—C5	120.7 (2)	H12A—C12—H12B	109.5
C1—C6—C7	113.0 (2)	C10—C12—H12C	109.5
C5—C6—C7	126.3 (2)	H12A—C12—H12C	109.5
O3—C7—N1	123.9 (2)	H12B—C12—H12C	109.5
			
O2—S1—N1—C7	−116.8 (2)	S1—C1—C6—C7	−1.2 (3)
O1—S1—N1—C7	112.4 (2)	C4—C5—C6—C1	0.9 (4)
C1—S1—N1—C7	−1.3 (2)	C4—C5—C6—C7	178.7 (2)
O2—S1—N1—C8	72.3 (2)	C8—N1—C7—O3	−6.7 (4)
O1—S1—N1—C8	−58.4 (2)	S1—N1—C7—O3	−177.5 (2)
C1—S1—N1—C8	−172.1 (2)	C8—N1—C7—C6	171.6 (2)
O2—S1—C1—C6	114.72 (19)	S1—N1—C7—C6	0.8 (3)
O1—S1—C1—C6	−110.00 (19)	C1—C6—C7—O3	178.5 (3)
N1—S1—C1—C6	1.38 (19)	C5—C6—C7—O3	0.6 (4)
O2—S1—C1—C2	−67.2 (3)	C1—C6—C7—N1	0.3 (3)
O1—S1—C1—C2	68.1 (3)	C5—C6—C7—N1	−177.6 (2)
N1—S1—C1—C2	179.5 (2)	C7—N1—C8—C9	97.6 (3)
C6—C1—C2—C3	0.9 (4)	S1—N1—C8—C9	−92.1 (3)
S1—C1—C2—C3	−177.0 (2)	C10—O5—C9—O4	1.2 (4)
C1—C2—C3—C4	−0.1 (4)	C10—O5—C9—C8	−179.3 (2)
C2—C3—C4—C5	−0.2 (4)	N1—C8—C9—O4	−10.1 (4)
C3—C4—C5—C6	−0.2 (4)	N1—C8—C9—O5	170.5 (2)
C2—C1—C6—C5	−1.3 (4)	C9—O5—C10—C12	−82.6 (3)
S1—C1—C6—C5	176.86 (19)	C9—O5—C10—C11	155.5 (2)
C2—C1—C6—C7	−179.3 (2)		

### Hydrogen-bond geometry (Å, º)

**Table:**

*D*—H···*A*	*D*—H	H···*A*	*D*···*A*	*D*—H···*A*
C8—H8*B*···O3^i^	0.99	2.27	3.236 (3)	166
C10—H10···O3^ii^	1.00	2.42	3.245 (3)	140

Symmetry codes: (i) −*x*+3/2, *y*+1/2, −*z*+3/2; (ii) *x*+1, *y*, *z*.
